# The 6q25.1 rs2046210 polymorphism is associated with an elevated susceptibility to breast cancer: A meta‐analysis of 261,703 subjects

**DOI:** 10.1002/mgg3.553

**Published:** 2019-01-28

**Authors:** Tie‐feng Jin, Wen‐ting Zhang, Zhen‐feng Zhou

**Affiliations:** ^1^ The Second Clinical Medical College Zhejiang Chinese Medical University Hangzhou, Zhejiang China; ^2^ Department of Anesthesiology Zhejiang Provincial People's Hospital Hangzhou, Zhejiang China

**Keywords:** 6q25.1, breast cancer, meta‐analysis, rs2046210 polymorphism

## Abstract

**Background:**

Several genome‐wide association studies already explored the associations between 6q25.1 rs2046210 polymorphism and breast cancer (BC), but the results of these studies were not consistent. Thus, we conducted a meta‐analysis of relevant studies to better analyze the effects of rs2046210 polymorphism on individual susceptibility to BC.

**Methods:**

PubMed, Web of Science, and Embase were searched for eligible studies. Odds ratios (ORs) and 95% confidence intervals (CIs) were calculated.

**Results:**

Totally 21 studies with 261,703 subjects were analyzed. A significant association with BC was observed for the rs2046210 polymorphism in GG versus GA +AA (dominant comparison, *p* < 0.0001, OR = 0.78, 95% CI 0.73–0.83), AA versus GG + GA (recessive comparison, *p* < 0.0001, OR = 1.21, 95% CI 1.18–1.24), GA versus GG + AA (overdominant comparison, *p* < 0.0001, OR = 1.12, 95% CI 1.08–1.16), and G versus A (allele comparison, *p* < 0.0001, OR = 0.86, 95% CI 0.82–0.89). Further subgroup analyses yielded similar positive results in both Asians and Caucasians.

**Conclusion:**

In summary, our findings suggested that the rs2046210 polymorphism may serve as a potential genetic biomarker of BC in both Asians and Caucasians.

## 1 INTRODUCTION

Breast cancer (BC) refers to malignancy that develops from epithelial tissue of breast. It is the second most common cancer globally, and the most frequent cancer among females (Siegel, Ma, Zou, & Jemal, 2014). In spite of rapid progress in chemotherapy and minimally invasive surgery achieved in the last few decades, BC still ranks as the fifth most common cause of cancer‐related deaths in both sexes, and the primary cause of cancer‐related deaths in women (Ferlay et al., 2015). Despite its high prevalence, the pathogenesis of BC is still not fully understood. Although obesity, hormone replacement therapy, and radiation were identified as potential risk factors of developing BC (Sun et al., 2017; Winters, Martin, Murphy, & Shokar, 2017), the fact that not everyone exposed to above‐mentioned carcinogenic factors ultimately develop BC suggests that inherited factors are also involved in the development of BC.

Recently, a genome‐wide association study (GWAS) conducted by zheng et al found that the rs2046210 G/A polymorphism at 6q25.1 was significantly associated with an elevated susceptibility to BC in both Chinese and Europeans (Zheng et al., 2009). Since then, numerous genetic association studies were performed in diverse populations, with inconsistent results (Barzan et al., 2013; Dai et al., 2012; Garehdaghchi, Derakhshan, & Khaniani, 2016; Han et al., 2011). Therefore, we conducted a meta‐analysis of all relevant studies to better analyze the effects of rs2046210 polymorphism on individual susceptibility to BC.

## 2 MATERIAL AND METHODS

### 2.1 Literature search and inclusion criteria

The current meta‐analysis followed the Preferred Reporting Items for Systematic Reviews and Meta‐analyses (PRISMA) checklist (Moher, Liberati, Tetzlaff, Altman, & PRISMA group, 2009). PubMed, Web of Science, and Embase were searched for potentially eligible articles using the combination of following terms: “6q25.1,” “rs2046210,” “polymorphism,” “variant,” “variation,” “mutation,” “genotype,” “allele,” and “breast cancer.” We also reviewed the reference lists of all retrieved articles for other potentially eligible studies.

To test the research hypothesis of this meta‐analysis, included studies should meet all the following criteria: (a) case–control study about rs2046210 polymorphism and BC; (b) providing sufficient data for calculating odds ratios (ORs) and 95% confidence intervals (CIs); (c) full text in English available. Studies were excluded if one of the following conditions was fulfilled: (a) not related to rs2046210 polymorphism and BC; (b) pedigree studies; (c) case reports or case series. In the case of duplicate reports by the same authors, we only included the most recent study.

### 2.2 Data extraction and quality assessment

We extracted the following information from eligible studies: (a) name of the first author; (b) year of publication; (c) country and ethnicity of participants; (d) sample size; and (e) the genotypic distribution of rs2046210 polymorphism in cases and controls. The probability value (*p* value) of Hardy–Weinberg equilibrium (HWE) was also calculated.

We used the Newcastle–Ottawa scale (NOS) to evaluate the quality of eligible studies (Stang, 2010). The NOS has a score range of zero to nine, and studies with a score of more than seven were thought to be of high quality.

Two reviewers conducted data extraction and quality assessment independently. When necessary, we wrote to the corresponding authors for extra information. Any disagreement between two reviewers was solved by discussion until a consensus was reached.

### 2.3 Statistical analyses

In the current study, we performed statistical analyses by using Review Manager Version 5.3.3. We calculated ORs and 95% CIs to estimate potential associations between rs2046210 polymorphism and BC in dominant (GG vs. GA + AA), recessive (AA vs. GG + GA), additive (GA vs. GG + AA) and allele (G vs. A) models, and a *p* value of 0.05 or less was defined as statistically significant. Between‐study heterogeneities were evaluated by *I* ^2^ statistic. Random‐effect models (REMs) would be used for analyses if *I* ^2^ was >50%. Otherwise, analyses would be conducted with fixed‐effect models (FEMs). Subgroup analyses were subsequently carried out by ethnicity. Stabilities of synthetic results were tested in sensitivity analyses. Publication biases were assessed by funnel plots.

## 3 RESULTS

### 3.1 Characteristics of included studies

**Figure 1:**
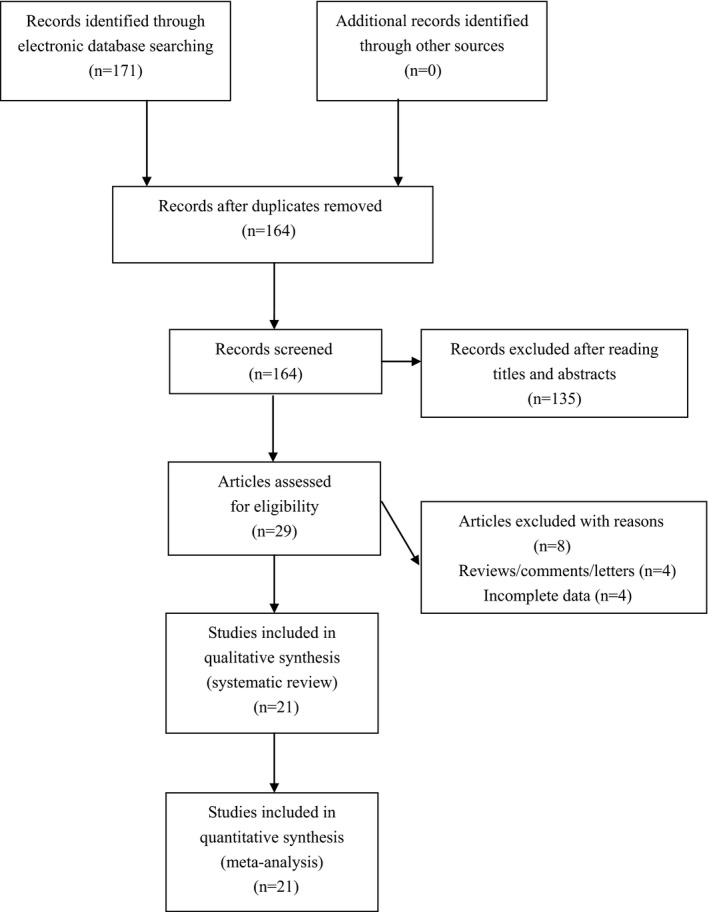
Flowchart of study selection for the present study

We found 171 articles by using our searching strategy. After excluding irrelevant and duplicate articles, 29 articles were retrieved for further evaluation. Another eight articles were subsequently excluded after reading the full text. Ultimately, a total of 21 eligible studies involving 131,785 cases and 129,918 controls were enrolled for analyses (see Figure 1). Characteristics of included studies were shown in Table 1.

**Table 1:** The characteristics of included studies for rs2046210 polymorphism and breast cancer

First author, year	Country	Ethnicity	Sample size	Genotype distribution		*p*‐Value for HWE	NOS score
				Cases	Controls		
Antoniou, 2011	UK	Caucasian	5,515/5,302	2,067/2,669/779	2,282/2,361/659	0.207	8
Barzan, 2013	Germany	Caucasian	311/960	NA	NA	NA	7
Barzan, 2013	Germany	Asian	984/2,206	NA	NA	NA	7
Cai, 2011	USA	Caucasian	4,373/3,885	1,689/2,097/587	1,576/1,836/473	0.081	8
Cai, 2011	USA	Asian	11,996/9,748	4,208/5,805/1,983	4,161/4,384/1,203	0.358	8
Campa, 2011	Germany	Caucasian	8,298/1,1543	3,322/3,796/1,180	4,904/5,161/1,478	0.037	8
Chan, 2012	Singapore	Asian	1,173/1,417	369/573/231	598/639/180	0.648	8
Dai, 2012	China	Asian	1,768/1,850	582/873/313	767/836/247	0.422	8
Garehdaghchi, 2016	Turkey	Caucasian	192/186	56/105/31	58/95/33	0.583	8
Han, 2011	Korea	Asian	3,251/3,493	1,260/1,565/426	1586/1531/376	0.820	8
He, Liu, Chen, Sun, Liu, & Shao 2016	China	Asian	254/339	89/122/43	139/144/56	0.076	8
Hein, 2012	Germany	Caucasian	54,298/40,904	22,357/24,190/6,751	21,823/21,751/5,520	0.366	8
Hein, 2012	Germany	Asian	2,983/2,334	1,041/1,455/487	1,020/1,048/266	0.897	8
Huo, 2012	USA	Mixed	1,509/1,383	NA	NA	NA	7
Jiang, 2011	China	Asian	493/510	NA	NA	NA	7
Kim, 2012	Korea	Asian	6,273/5,871	NA	NA	NA	7
Mulligan, 2011	UK	Caucasian	4,646/4,352	1,732/2,255/659	1,886/1,919/547	0.088	8
Ruiz‐Narváez , 2012	USA	Mixed	1,191/1,941	NA	NA	NA	7
Stacey, 2010	Iceland	Caucasian	7,899/11,234	NA	NA	NA	7
Stacey, 2010	Iceland	Asian	1,126/1,118	NA	NA	NA	7
Stacey, 2010	Iceland	Mixed	1,151/934	NA	NA	NA	7
Stevens, 2011	USA	Mixed	27,07/1,385	NA	NA	NA	7
Wang, 2012	China	Asian	1,062/1,072	349/517/196	447/475/150	0.189	8
Zheng, 2009	USA	Mixed	810/1,784	NA	NA	NA	7
Zheng, 2009	USA	Asian	6,472/3,962	2,162/3,208/1,102	1,614/1,812/536	0.443	8
Zheng, 2009	USA	Caucasian	1,591/1,466	614/761/216	617/690/159	0.098	8
Zhou, 2015	China	Asian	459/549	159/217/83	223/252/74	0.834	8

Note

HWE: Hardy–Weinberg equilibrium; NA: Not available; NOS: Newcastle–Ottawa scale.

### 3.2 Overall and subgroup analyses

Totally 261,703 subjects were analyzed. A significant association with BC was observed for the rs2046210 polymorphism in GG versus GA + AA (dominant comparison, *p* < 0.0001, OR = 0.78, 95% CI 0.73–0.83), AA versus GG +GA (recessive comparison, *p* < 0.0001, OR = 1.21, 95% CI 1.18–1.24), GA versus GG + AA (overdominant comparison, *p* < 0.0001, OR = 1.12, 95% CI 1.08–1.16), and G versus A (allele comparison, *p* < 0.0001, OR = 0.86, 95% CI 0.82–0.89). Further subgroup analyses yielded similar positive results in both Asians and Caucasians (see Table 2).

**Table 2:** Results of overall and subgroup analyses for rs2046210 polymorphism and breast cancer

Population	Sample size	Dominant comparison			Recessive comparison			Overdominant comparison			Allele comparison		
		*p* Value	OR (95% CI)	*I* ^2^ statistic	*p* Value OR	(95% CI)	*I* ^2^ statistic	*p* Value	OR (95% CI)	*I* ^2^ statistic	*p* Value	OR (95% CI)	*I* ^2^ statistic
Overall	131,785/129,918	**<0.0001**	**0.78 (0.73–0.83)**	88%	**<0.0001**	**1.21 (1.18–1.24)**	72%	**<0.0001**	**1.12 (1.08–1.16)**	62%	**<0.0001**	**0.86 (0.82–0.89)**	89%
Asian	38,294/34,469	**<0.0001**	**0.72 (0.70–0.75)**	0%	**<0.0001**	**1.39 (1.32–1.46)**	13%	**<0.0001**	**1.16 (1.12–1.20)**	0%	**<0.0001**	**0.79 (0.73–0.84)**	87%
Caucasian	86,123/88,022	**<0.0001**	**0.86 (0.82–0.91)**	71%	**<0.0001**	**1.14 (1.11–1.18)**	0%	**0.001**	**1.08 (1.03–1.14)**	64%	**<0.0001**	**0.91 (0.88–0.94)**	64%

Notes

CI: Confidence interval; NA: Not available; OR: Odds ratio.

The values in bold represent there are statistically significant differences between cases and controls.

### 3.3 Sensitivity analyses

We conducted sensitivity analyses by eliminating one individual study each time. The significant associations detected in pooled analyses remained unchanged in all comparisons, which suggested that our findings were statistically stable.

### 3.4 Publication biases

We used funnel plots to evaluate potential publication biases. The shape of funnel plots was symmetry for every comparison, which indicated that severe publication biases were unlikely.

## 4 DISCUSSION

To the best of our knowledge, this is so far the most comprehensive meta‐analysis about rs2046210 polymorphism and BC. The pooled analyses revealed that the rs2046210 polymorphism was significantly associated with BC in both Asians and Caucasians. The stabilities of synthetic results were evaluated by sensitivity analyses, and no alterations of results were observed in any comparisons, which suggested that our findings were statistically stable. As for evaluation of heterogeneities, significant heterogeneities were detected in every comparison of overall analyses, and thus all analyses were performed with REMs. But in further subgroup analyses, a reduction tendency of heterogeneity was found for Asians, which suggested that differences in ethnicity could partially explain observed heterogeneities between studies.

There are several points that worth noting about this meta‐analysis. Firstly, the 6q25.1 rs2046210 polymorphism is located within 1‐Mb upstream of *ESR1*, the encoder of ERα. Since it was evident the binding of estrogen and ERα could result in increased proliferation of normal and cancerous breast epithelial cells (Ali & Coombes, 2000; Russo & Russo, 2006), it is possible that rs2046210 polymorphism may alter the expression level of *ESR1 *and consequentially influence individual susceptibility to BC. Secondly, the etiology of BC is extremely complex, and as a consequence, to better elucidate potential roles of genetic variations in BC, we strongly recommend future studies to conduct haplotype analyses and investigate potential gene–gene interactions.

Some limitations of this meta‐analysis should also be noted when interpreting our findings. First, our pooled analyses were based on unadjusted estimations due to lack of raw data, but we have to admit that failure to perform further adjusted analyses may impact the reliability of our findings (Xie, Shi, & Liu, 2017). Second, heterogeneities between studies remained significant in certain subgroup comparisons, especially for Caucasians, which suggested that the inconsistent results of included studies could not be fully attributed to ethnicity, and differences in other unmeasured characteristics of participants may also contribute to heterogeneities (Shi, Xie, Jia, & Li, 2016). Third, associations between rs2046210 polymorphism and BC may also be modified by gene–gene and gene–environmental interactions. However, most studies did not consider these potential interactions, which impeded us to conduct relevant analyses (Shi et al., 2015). Considering the above‐mentioned limitations, our findings should be interpreted with caution.

## 5 CONCLUSIONS

In summary, our meta‐analysis suggested that the rs2046210 polymorphism may serve as a potential genetic biomarker of BC in both Asians and Caucasians. However, further well‐designed studies are still needed to confirm our findings.

## 6 INFORMED CONSENT

For this type of study formal consent is not required.

## ETHICAL APPROVAL

This article does not contain any studies with human participants or animals performed by any of the authors.

## CONFLICT OF INTEREST

The authors declare that they have no conflict of interest.

## AUTHORS' CONTRIBUTIONS

Tie‐feng Jin and Zhen‐feng Zhou conceived of the study, participated in its design. Tie‐feng Jin and Wen‐ting Zhang conducted the systematic literature review. Tie‐feng Jin and Wen‐ting Zhang performed data analyses. Tie‐feng Jin and Zhen‐feng Zhou drafted the manuscript. All authors have read and approved the final manuscript.
